# Validation of verbal autopsy methods using hospital medical records: a case study in Vietnam

**DOI:** 10.1186/s12874-018-0497-7

**Published:** 2018-05-18

**Authors:** Hong Thi Tran, Hoa Phuong Nguyen, Sue M. Walker, Peter S. Hill, Chalapati Rao

**Affiliations:** 1 Faculty of Fundamental Sciences, Hanoi University of Public Health, Hanoi, Vietnam; 2 School of Public Health, University of Queensland, Brisbane, Australia; 3 Family Medicine Department, Hanoi Medical University, Hanoi, Vietnam; 4 School of Public Health and Social Work, Queensland University of Technology, Brisbane, Australia; 5 National Centre for Health Information Research and Training, Queensland University of Technology, Brisbane, Australia; 6 Department of Global Health, Research School of Population Health, ANU College of Health and Medicine, Australian National University, Canberra, Australia

**Keywords:** Validation of verbal autopsy, Causes of death, Validity, Medical record, Hospital data, Verbal autopsy, Health information, Quang Ninh, Vietnam

## Abstract

**Background:**

Information on causes of death (COD) is crucial for measuring the health outcomes of populations and progress towards the Sustainable Development Goals. In many countries such as Vietnam where the civil registration and vital statistics (CRVS) system is dysfunctional, information on vital events will continue to rely on verbal autopsy (VA) methods. This study assesses the validity of VA methods used in Vietnam, and provides recommendations on methods for implementing VA validation studies in Vietnam.

**Methods:**

This validation study was conducted on a sample of 670 deaths from a recent VA study in Quang Ninh province. The study covered 116 cases from this sample, which met three inclusion criteria: a) the death occurred within 30 days of discharge after last hospitalisation, and b) medical records (MRs) for the deceased were available from respective hospitals, and c) the medical record mentioned that the patient was terminally ill at discharge. For each death, the underlying cause of death (UCOD) identified from MRs was compared to the UCOD from VA. The validity of VA diagnoses for major causes of death was measured using sensitivity, specificity and positive predictive value (PPV).

**Results:**

The sensitivity of VA was at least 75% in identifying some leading CODs such as stroke, road traffic accidents and several site-specific cancers. However, sensitivity was less than 50% for other important causes including ischemic heart disease, chronic obstructive pulmonary diseases, and diabetes. Overall, there was 57% agreement between UCOD from VA and MR, which increased to 76% when multiple causes from VA were compared to UCOD from MR.

**Conclusions:**

Our findings suggest that VA is a valid method to ascertain UCOD in contexts such as Vietnam. Furthermore, within cultural contexts in which patients prefer to die at home instead of a healthcare facility, using the available MRs as the gold standard may be meaningful to the extent that recall bias from the interval between last hospital discharge and death can be minimized. Therefore, future studies should evaluate validity of MRs as a gold standard for VA studies in contexts similar to the Vietnamese context.

**Electronic supplementary material:**

The online version of this article (10.1186/s12874-018-0497-7) contains supplementary material, which is available to authorized users.

## Background

Verbal autopsy (VA) is a method of ascertaining cause of death (COD) from information on signs/symptoms and circumstances preceding death through interviewing the deceased’s caretakers [1]. VA is used as a research tool for longitudinal population studies, intervention research and epidemiological studies. It also has become a source of COD statistics at population level in some countries, providing cause-specific mortality data that can be used in priority setting for planning and policy formulation [2, 3]. VA is the best available approach to describe the causes of death at community level or population level in countries where most deaths occur at home and without medical evidence [4].

Information on COD is crucial for measuring the health outcomes of populations and progress toward the Sustainable Development Goals [2, 5]. In many low and middle-income countries including Vietnam, where the civil registration and vital statistics (CRVS) systems are dysfunctional [6–8], such information will continue to rely on VA methods [9–11]. During the past 15 years, VA methods have been used in number of studies, projects and the health and demographic surveillance system (HDSS) in Vietnam to identify mortality patterns in specific populations [8, 12–18]. In order to establish the utility of the information on COD derived from a VA study, the validity of COD derived from VA in Vietnam needs to be assessed.

Validation is a process that compares an underlying cause of death (UCOD) derived from the VA with a reference UCOD for the same death. The reference UCOD can be derived from pathological autopsy which is considered as a “gold standard”, or from clinical records which are considered as the next best alternative [19]. Chandramohan suggested that hospital diagnosis of COD which is based on defined laboratory and clinical criteria are “the only useful gold standards” available at present for validating VAs [20]. A number of VA validation studies conducted in other countries used the hospital medical records (MRs) of inpatient deaths as reference diagnoses [19, 21, 22]. However, due to cultural beliefs and traditional issues among the Vietnamese, it is a common practice for terminally ill patients to return home for the final stages of their lives [8, 11, 17]. The number of hospital deaths is very low [23], which hampers the implementation of a validation study. However, we believe that the hospital MRs of patients discharged in a terminally ill condition could provide useful information for diagnosing COD. Therefore, in order to carry out a validation study in the Vietnamese context, we adjusted the method to suit the circumstances in the country.

We conducted this study to assess the validity of COD identified from the VA methods which had been implemented in a recent study in Vietnam. Building on empirical experiences during the study fieldwork, this paper also makes recommendations on some methodological issues for implementing hospital based validation studies in Vietnam.

## Methods

### Study setting and protocol

We designed this validation study based upon the information extracted from a recent VA study in 12 communes in Quang Ninh province. A detailed description of this VA study are available elsewhere [24]. Complete VA interviews for 670 deaths that occurred between 1/1/2014 to 31/12/2014 were successfully conducted, using a Vietnamese adapted version of international standard VA questionnaires. The adapted questionnaires have been used in a previous mortality surveillance project and some other studies in Vietnam [8, 25–27]. Interviewers were commune health workers and village health workers who received training in administering VA interviews. Multiple CODs and UCOD for each death were derived from completed VA questionnaires according to international standards and rules.

The VA questionnaire recorded the place of death, including the name of the hospital where death occurred. In case of deaths outside hospital, the questionnaire asked and recorded the last hospital attended during the terminal illness, and the interval (number of days and/or months) between hospital discharge and death.

Of 670 deaths in Quang Ninh province, cases which were included in the validation study needed to meet the following criteria:

1. Criterion 1: Deaths in hospitals. If the deceased did not die in a hospital/health facility then deaths within 30 days of discharge after last hospitalization (we assumed that the causes of death are very likely to be related to the discharge diagnosis and the information recorded in the MRs); and
2. Criterion 2: MRs for the deceased were available from the three hospitals which had the highest number of cases that satisfied criterion 1; and
3. Criterion 3: These MRs included documentation of the condition of the patient at discharge as “*died*” or “*severe, the family wish to take the patient home*”.

### Identification of MRs and MR abstraction

At first, the name, date of birth, sex and address were used to determine if the case found in the hospital database was the same as the case identified from the VA questionnaire. Subsequently, the complete MRs were retrieved from the archives, including documents from previous admissions in the same hospital. The MRs pertaining to the final admission were used as main source for MRs abstraction. The other records were reviewed to get additional information.

In each hospital, trained hospital nurses abstracted relevant details from the MRs onto a specific study data abstraction form (see Additional file 1). Accordingly, information about medical history of the patients, signs, symptoms, presenting illness and clinical events during hospitalization which culminated in either death or discharge of the patient, any relevant investigation results and diagnoses as well as laboratory tests performed were extracted onto the abstraction form.

### UCOD identification and coding

In the next step, a senior trained physician with over 20 years of clinical experience reviewed the MRs abstracts and completed a Death Certificate (see Additional file 2) for each death, following the International Medical Certificate of cause of death format [28]. This form has two parts which allows documentation of the direct, antecedent and underlying causes of death to be recorded in part I and other conditions that may have contributed to the death to be recorded in part II. The completed death certificates then were coded by a senior trained coder using ICD-10 [29]. It is noted that all causes recorded in the death certificates were coded. Then the UCOD was assigned following the current international mortality coding rules [28]. According to the WHO standard definition, the UCOD is “the disease or injury which initiated the train of morbid events leading directly to death, or the circumstances of the accident or violence which produced the fatal injury” [28].

The accuracy of UCOD assignment was confirmed and reviewed by an independent expert coder using the most updated version of the Iris software (version 5) [30], using the “MUSE” function. The cause used for comparison for validating the VA was the UCOD. All people who participated in the data collection in hospitals, including the physician who reviewed the MRs abstraction and the ICD-10 coder, were blinded to the VA data to avoid information bias.

### Data management and analysis

For efficiency in analyses, ICD codes for all COD were aggregated according to the WHO General Mortality Tabulation Condensed List I, comprising 103 categories of ICD-10 codes [31]. For assessing the validity of VA diagnoses, the UCOD identified from the MRs review were compared with the UCOD identified from the VA for each case. As a first step in the analysis, the percentage of agreement between underlying causes from MRs and VA were computed. Subsequently, we used the MRs diagnoses as the reference value to compute the sensitivity, specificity and positive predictive value (PPV) of the UCOD diagnosed from VA [19, 20, 22, 32–38]. Measures of validity were computed for conditions known to be among the leading causes of death in Vietnam [8]. We categorised validity as good if sensitivity was above 75%, average if sensitivity lay between 50 to 75%, and low if sensitivity is less than 50%, similar to ratings used in other studies [39, 40]. Standard errors were computed for these proportions, and used to develop 95% confidence intervals for each of these values.

Validity of VA methods was also analysed by age of the deceased broadly categorized into 25-69 years and 70 and above. It is generally perceived that VA interviews are less informative for deaths in the elderly (i.e above 70 years), hence this age group analysis was undertaken to observe any variations in levels of validity for specific causes by age. In a subset of cases where the UCOD from VA did not match that from the MRs, we provided a descriptive summary of all reported COD from the MR.

## Results

### Description of the study population

**Fig. 1:**
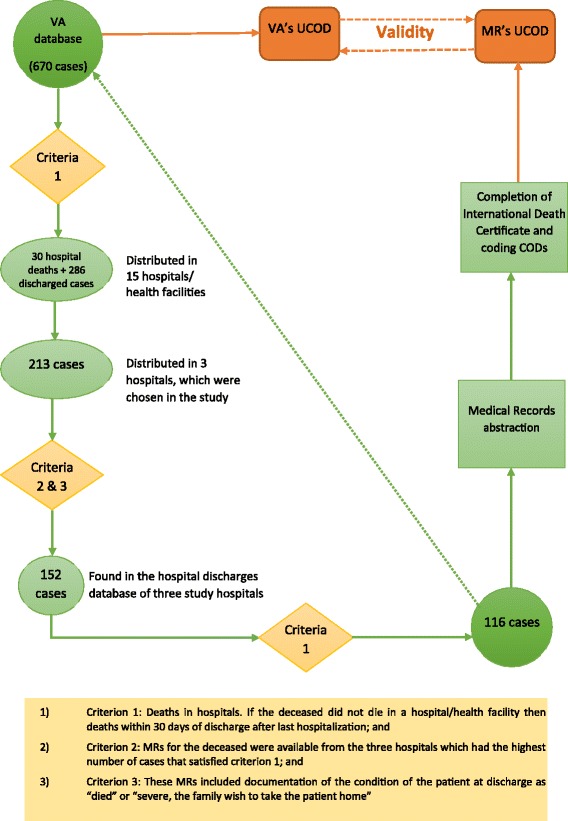
Study population and study protocol for validation of VA methods

The description of the selection of study population is shown in Fig. 1.

Of the 213 cases that were identified by the VA study to have been hospitalised in the three study hospitals, only 152 cases were found in the hospital records. Information on the status of patients on discharge (1-Recovered, 2-Severe, the family wish to take the patient home, 3-Referred, and 4-Died); and the date of admission and the date of discharge was reviewed. As part of this step, selection criterion 1 for the study sample was re-checked for all cases. It was found that there were 36 cases for which the MRs showed that they were discharged more than 30 days prior to the death, and hence these cases were excluded from the study sample. The total number of eligible cases for the validation study was therefore 116 deaths, accounting for only 54.5% of the initially extracted list (116/213). Table 1 shows more details of the study sample at each step of the selection process.

**Table 1:** Summary of the study sample by hospital

Name of the hospital	The initially extracted list from VA database	MRs found	Eligible cases	Eligible cases compared to the initially extracted list
	(n1)	(n2; n2/n1)	(n; n/n2)	(n/n1)
1. Quang Ninh General hospital	94	67; 71.3%	51; 76.1%	54.3%
2. Bai Chay district Hospital	47	37; 78.7%	32; 86.5%	68.1%
3. Vietnam-Swiss Uong Bi Hospital	72	48; 66.7%	33; 68.8%	45.8%
Total	213	152; 71.4%	116; 76.3%	54.5%

In our final study sample (116 cases), 70% of the deceased were male and 30% were female. Almost all were adult deaths with ages which ranged from 28 to 96 years old. About 45% of deaths were in the age group 70 years and older, 38% in the 50-69 year age group and 16% in the age group of 25-49 years. There was a single one-year-old child death which accounted for 0.9% of the sample. Only 5% of the deceased died in a hospital. 95% of them were discharged in a severe condition and then died within 30 days at their home.

**Fig. 2:**
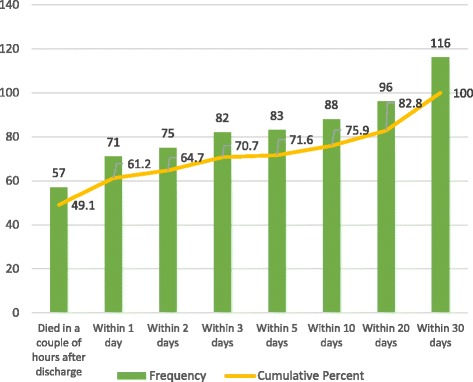
Interval between patient discharge and death

Figure 2 describes the duration of the interval from the date of the patient’s discharge to the date of death. About 50% died on the same day as they were discharged from the hospital; 61% died within 24 hours (one day); over 70% of patients died within three days; 76% of them died within 10 days after the family took them home.

### Validity of the UCOD from VA

At first, we calculated the percentage agreement on UCOD between VA and the corresponding MRs. As can be seen in Table 2, there were about 57% of cases in which both sources assigned the same UCOD. There were 22 cases (19%) in which the UCOD from VA was different to that from the MRs, but the condition recorded as the UCOD in the VA was one of the multiple causes recorded in the MRs.

**Table 2:** Agreement on UCOD between VA and hospital MRs

	Age 25-69 (n; %)	Age 70+ (n; %)	Total (n; %)
UCOD from VA is the same as UCOD from MR	38 (60%)	28 (54%)	66 (57%)
UCOD from VA is one of multiple causes recorded in MR	7 (11%)	14 (27%)	22 (19%)
UCOD by VA is different from any cause in MR	18 (29%)	10 (19%)	28 (24%)
Total	63	52	116

To further understand the validity of the specific causes diagnosed by VA, sensitivity, specificity and PPV were calculated for leading causes. As presented in the Table 3, specificity scores were very high for all causes (from 91 to 100%), which means VA is very good at not assigning a cause for a patient who really did not die of that cause. One possible reason for this high specificity is that for each cause, the number of true negatives from both sources of the study sample will always be high, owing to them being truly from other causes.

**Table 3:** Validity of the VA diagnosis on cause of death

Underlying cause of death	ICD code	True positive	True negative	Cases diagnosed from MRs	Cases diagnosed from VA	Sensitivity, (95% CI)	Specificity, (95% CI)	PPV, (95% CI)
Cancer								
1. Lung cancer	C34	8	108	8	8	100	100	100
2. Liver cancer	C22	6	108	6	8	100	98 (96-100)	75 (45-100)
3. Colon and rectum cancers	C18-C21	4	112	4	4	100	100	100
4. Mouth and oropharynx cancers	C00-C14	3	112	4	3	75 (33- 100)	100	100
5. Oesophagus cancer	C15	3	113	3	3	100	100	100
6. Stomach cancer	C16	2	114	2	2	100	100	100
7. Pancreas cancer	C25	2	114	2	2	100	100	100
Cadiovascular diseases								
8. Stroke	I60-I69	14	89	18	23	78 (59-97)	91 (85-97)	61 (41-81)
9. Ischaemic heart disease (IHD)	I20-I25	3	105	9	5	33 (3-64)	98 (96-100)	60 (17-100)
Other non-communicable diseases and external causes								
10. Diabetes mellitus	E10-E14	2	106	5	7	40 (0-83)	95 (92-99)	29 (0-62)
11. Cirrhosis of liver	K70, K74	2	110	5	3	40 (0-83)	99 (97-100)	67 (13-100)
12. Chronic obstructive pulmonary diseases (COPD)	J40-J44	1	109	7	1	14 (0-40)	100	100
13. Road traffic Injury	V01-V04, V06, V09-V80, V87, V89, V99	4	112	4	4	100	100	100
Communicable diseases								
14. HIV/AIDS	B20-B24	2	113	3	2	67 (13-100)	100	100
15. Pneumonia	J12-J18	2	102	6	10	33 (0-71)	93 (88-98)	20 (0-45)
Other causes		8		30	31			
Total		66		116	116			

VA performed very well in diagnosing almost all major site-specific cancers (such as lung cancer, liver cancer, colon and rectum cancer) and road traffic accidents with scores of 75 to 100% for sensitivity and PPV. In the cardiovascular diseases group, the validity of VA varied among different causes. For stroke, sensitivity indicated that VA could capture nearly 80% of patients whose cause of death was actually stroke. However, the PPV indicates that VA tended to over-diagnose deaths due to stroke, with only 61% of VA diagnoses of stroke being confirmed by the MRs review.

Several other causes were also found to have low sensitivity (below 50%). VA correctly diagnosed only 33% deaths due to ischemic heart disease (IHD), 40% deaths due to cirrhosis of liver, 40% deaths due to diabetes, 33% deaths due to pneumonia and only 14% deaths due to COPD. We conducted a similar analysis of validity of VA diagnoses for age groups 25-69 and 70+ years. However, there was no difference between the two age groups regarding sensitivity, specificity and PPV scores for leading CODs (see Additional file 3).

As mentioned in Table 1, we found 19% of cases where the UCOD from VA was one of causes listed on the MRs death certificate. We further analysed these 22 cases to better understand the misclassification patterns of underlying causes from VA, Table 4 presents information on multiple COD derived from available information in hospital MRs as well as from VA, according to the format of the international death certificate.

**Table 4:**
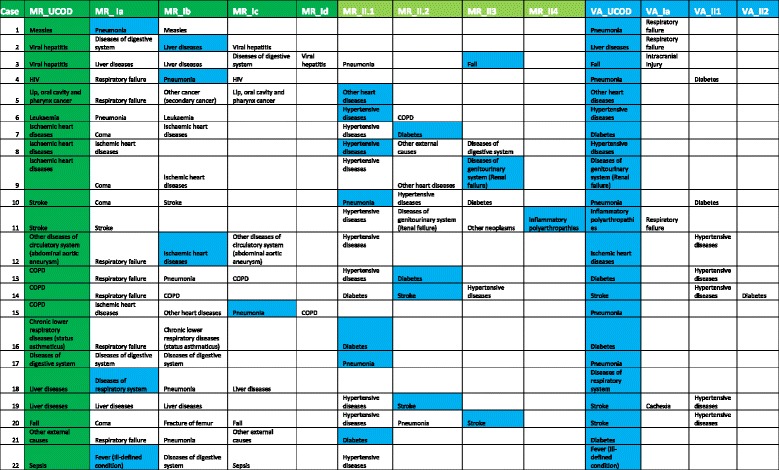
Description of multiple causes of death for 22 cases

MR_UCOD is the underlying COD derived from information in MR. MR_Ia is the MR’s COD recorded in Part I(a) of death certificate. MR_Ib is the MR’s COD recorded in Part I(b). Similar to MR_Ic and MR_Id. MR_II.1, MR_II.2, MR_II.3, MR_II.4 are the MR’s COD recorded in the part II of death certificate. VA_UCOD is the underlying COD by VA. VA_Ia is the VA’s COD recorded in Part I (a). Similar explanation for VA_II.1 and VA_II.2. The “green data” indicated the UCOD which was assigned base on MRs while the “blue data” indicated the UCOD which was assigned base on VA methods. Some “blue data” was highlighted in other selected cells inside the table, which indicates that the cause/condition assigned as UCOD from VA is one of causes recorded in MRs (but not UCOD)

In about 32% (7/22) of cases, the UCOD from VA was either the direct cause or one of the antecedent causes identified from hospital MRs by the physician (cases 1, 2, 4, 12, 15, 18 and 22). The VA respondents for these cases might have only remembered the cause/conditions/signs which directly led to the death. For example, for case number 1 (Table 4), pneumonia was the direct cause which was a consequence of the UCOD, measles. However, the VA respondent might not have reported the skin rash which is the only differentiating symptom indicative of measles as the underlying condition that caused pneumonia. Hence, based on the reported signs and symptoms, a diagnosis of pneumonia was derived, and this is a reasonable expectation of cause of death assignment from VA. Eventually, the identification of pneumonia by the VA in this case underscores the public health utility of this method for ascertaining causes of death. Table 4 also shows that in 68% of cases (15/22) the UCOD from VA was a contributory cause recorded in Part II of the death certificate from the MRs. Most of these causes were non-communicable diseases such as hypertensive diseases, diabetes mellitus, or stroke. These results also indicate the usefulness of VA in correctly identifying these major co-morbidities, and hence justifying the value of VA in settings experiencing a growing epidemic of non-communicable diseases, as in Vietnam. Therefore, establishing the validity of VA in determining such comorbidity through ascertaining multiple causes of death also adds value to the overall public health utility of VA.

## Discussion

To our knowledge, this is the first known study to assess the validity of VA methods in Vietnam using hospital records to provide a diagnostic benchmark. Previous studies have identified the utility of VA for diagnosing deaths from stroke [11] and injuries [38], but did not employ specific comparison of VA diagnoses with reference standards in order to measure validity. Establishing the validity of VA methods is important when considering to use these methods on a routine basis to improve the availability and quality of mortality data in the country.

### The reference diagnosis

Previous VA validation studies which have been conducted in other countries used the UCOD determined by physician review of hospital MRs of inpatient deaths as the reference diagnosis for validation [21, 22, 41, 42]. In Vietnam, VA methods are routinely used in three Demographic and Health Surveillance Systems and in a number of projects/studies to measure population level cause-specific mortality. However, non-availability of a “gold standard” reference diagnosis for the underlying cause has been described as a constraint for the implementation of a validation study [19]. This study demonstrated a potential solution for overcoming this constraint in the Vietnam context. In designing this study, we first noted that from the VA database of 670 deaths in Quang Ninh province, we found only 30 cases (about 5%) who died in a hospital/health facility. However, about 70% of the deceased had been hospitalised during their terminal illness [24]. Hence, we chose to use the MRs of patients who had been discharged from hospital within 30 days prior their death, as the source for reference diagnoses to validate the COD from VA.

### Validity of VA and potential for its improvement

In this study we found that for the UCOD comparison across all cases, there was 57% agreement between VA and physician review of hospital MRs. This percentage of agreement on cause-specific codes is much higher than found in a study conducted in Malawi, which reported only 26.2% agreement [41]. When taking into account the comparison across multiple causes, the underlying cause from VA matched with one of the multiple causes in the MRs in 76% of cases. These overall findings confirmed the value of VA methods in identifying the COD for deaths which occur outside hospitals in Vietnam.

Despite these high levels of agreement as well as good sensitivity scores for some causes of death, the relatively lower sensitivity of VA UCOD diagnoses for several important conditions including ischaemic heart disease, COPD, diabetes and pneumonia is a cause for concern. Similar findings of low sensitivity scores (< 50%) for these specific causes of death have also been observed in other verbal autopsy validation studies in China [19, 40], Malaysia [43] and Thailand [42]. To some extent, the analysis of multiple causes of death as presented in Table 4 has improved our understanding of misclassification patterns that result in low sensitivity scores for these conditions as underlying causes. In view of the potential for several of these conditions to be co-existent in many instances, the tools and processes employed for VA data collection, diagnosis and coding should routinely facilitate the reporting and analysis of multiple causes. While this would enhance the utility of VA data for both descriptive and analytical epidemiology, multiple cause analysis will also strengthen the assessment of validity of VA diagnoses in future studies.

Obtaining medical records or at least discharge diagnoses for terminally ill patients could be an important source of information to strengthen cause of death ascertainment for such events. Such information is particularly relevant for conditions that need specific clinical diagnostic tests or imaging investigations. If every discharged patient is provided with a discharge summary relating to their hospitalisation, this would provide valuable information for ascertaining the COD, in the case of a VA interview. The interviewers could ask the family member to show those documents and therefore important information may be obtained.

### Limitations

A key limitation of this study is the small sample size which resulted in the large ranges of 95%CI of sensitivity and PPV values for specific causes. The small sample size critically hampers more detailed analysis of validity for a wider range of causes, as well as for any variations in cause-specific validity across gender and rural/urban areas. To start with, the initial sampling frame for this study was limited to the sample of deaths associated with selected hospitals from one province, owing to restrictions in terms of costs, manpower, and other logistical factors. Further, as mentioned previously, there were losses to follow up during the study implementation. For instance, only 71.4% of the 213 eligible cases reported in the VA study to have accessed treatment from the three hospitals were actually traced in the hospital records. The remaining 28.6% cases could not be matched according to the full name, sex, date of birth and address of the deceased. There are probably two reasons for this phenomenon. The first may be the issue of misinformation or miscommunication of relevant identification details by the patient (or the patient’s relatives) with the hospital’s administration/reception at admission. In many cases in Vietnam, the elderly live with their children in another province, the hospital may record their current address, while the VA records their original home address [8], resulting in a mismatch. The second reason may due to incorrect information in the VA interview in response to the question on last treatment accessed before death. In some instances, individuals receive treatment as an outpatient in several different clinics or hospitals for the same illness, and at the VA interview, the deceased’s relative might have provided an incorrect health facility name as the site for last hospitalization.

There was an additional 24% (36 cases) of cases that were excluded since the actual date of hospitalization reported at the VA interview was incorrect. These individuals had actually been discharged more than 30 days prior to death, and hence had to be dropped from the study. Eventually, the final number of 116 study cases accounted for only 55% of the 213 cases in the initial extracted list. These losses to follow up further limited the study sample size but were considerably less than the 75% losses observed in a validation study which was conducted in Ethiopia with a similar approach to trace medical records of community reported deaths in hospital records [21]. Such potential losses to study samples and the reasons for the same should be anticipated in establishing the study design and sample size for future VA validation studies in Vietnam and other similar settings.

A potential limitation of the approach to use hospital discharge records as a data source for ascertaining causes of death is that there may be cause(s) other than the discharge diagnosis that could have resulted in the death. The potential for such instances would increase with the length of the interval between discharge and death. Further research evidence would be required to identify the most suitable cut-off time for the use of reference diagnoses from the medical records for discharged patients in VA validation studies.

## Conclusion

Our findings suggest that VA is a valid method to ascertain UCOD in contexts such as Vietnam. Furthermore, within cultural contexts in which patients prefer to die at home instead of a healthcare facility, using the available MRs as the gold standard may be meaningful to the extent that recall bias from the interval between last hospital discharge and death can be minimized. Therefore, future studies should evaluate validity of MRs as a gold standard for VA studies in contexts similar to the Vietnamese context.

## Additional files

Additional file 1: Medical record abstraction form. This supplementary document provides the form which was used to abstract information from medical records. (PDF 215 kb)

Additional file 2: Death Certificate. This supplementary document provides the International form of medical certificate of cause of death. (PDF 266 kb)

Additional file 3: Validity by age groups. This supplementary document included two tables which describe the calculation of validity of the VA diagnosis on causes of death by age 25-69 years and 70+ years. (PDF 82 kb)

## Abbreviations

COD: Causes of death
CRVS: Civil registration and vital statistics
DHSSs: Demographic health surveillance systems
IHD: Ischemic heart diseases
MRs: Medical records
UCOD: Underlying cause of death
VA: Verbal autopsy
