# Supplementary material for: Validation of verbal autopsy methods using hospital medical records: a case study in Vietnam
Source: BMC Med Res Methodol. 2018 May 18;18:43. doi: 10.1186/s12874-018-0497-7 (PMC5960129; doi:10.1186/s12874-018-0497-7)
Supplement: Supplementary file 1 — Medical record abstraction form. This supplementary document provides the form which was used to abstract information from medical records. (PDF 215 kb) [file 12874_2018_497_MOESM1_ESM.pdf]

Additional file 1. MEDICAL RECORD ABSTRACTION FORM

Study ID of the deceased: | | | | | | | | |

Hospital's ID:

____

Date:

____ /____ / ____

Part 1: PATIENT DEMOGRAPHICS

1.1 Paient's ID Number: | | | | | | | | |

1.2 Medical record storage number: | | | | | | | | |

1.3 Sex ☐ 1 Male ☐ 2 Female

1.4 Date of Birth: ____/___/___ 1.5 Date of Death: ___/___/_____
dd mm yyyy dd mm yyyy

1.5 Address of Deceased (*as detailed as possible*)

____

____

1.6 Case died in Hospital? ☐ 1 YES ☐ 2 NO

1.7 Date of Admission /____/____/____ 1.8. Date of Discharge /____/____/____
dd mm yyyy dd mm yyyy

1.9. Patient's condition at the discharge:

☐ 1 Recover ☐ 2 Severe (patients were taken home)

☐ 3 Referred ☐ 4 Died in the hospital

1.10. Diagnosis at discharge:

Main disease: ……………………………………………… ICD-10 code:………………………

Other diseases: ……………………………………………..ICD-10 codes: ……………………

1.11. If the patient died at the hospital, what is cause of death recorded by hospital?

____

Part 2: FEMALE DEATHS

☐ Not Applicable

2.1 Was this person pregnant ☐ 1 YES ☐ 2 NO

2.2 Did the death occur within 6 weeks of either a delivery or abortion?

☐ 1 YES ☐ 2 NO

2.3 If yes to either question:

Provide details – (include Date of Delivery, complications, single or multiple birth, child born alive etc)

Part 3: HISTORY, EXAMINATION, INVESTIGATION AND PROGRESS

3.1. The reasons for hospitalisation (for example: fever, cough, injury due to traffic accident. If the person hospitalised due to injury, describe how did it occur? Type of injuries):

3.2 Diseases history (Does the patient has any chronic diseases/conditions? Any current treatment for these diseases?, for how long? Any noted complications from the chronic condition? Was any surgery / invasive procedure performed on this person?,…):

3.3. Clinical case summary: (please write a brief description of the presenting illness and clinical events during hospitalization in chronological sequence, culminating in either death or discharge of the patient. Include any relevant investigation results and diagnoses, as recorded by treating physicians in the case record).

3.4 Provide details of all laboratory investigations performed, positive test results – include dates / test type and results

3.5 Provide details of all imaging studies performed, positive imaging results – include dates / study type and results

3.6 Describe any Histopathology results (cancers, liver and renal biopsies)

☐ Not Applicable

3.7. Is there a positive HIV test result on file ☐ 1 YES → __/ __ / __ ☐ 2 NO

THE END.
