# Supplementary material for: Validation of verbal autopsy methods using hospital medical records: a case study in Vietnam
Source: BMC Med Res Methodol. 2018 May 18;18:43. doi: 10.1186/s12874-018-0497-7 (PMC5960129; doi:10.1186/s12874-018-0497-7)
Supplement: Supplementary file 2 — Death Certificate. This supplementary document provides the International form of medical certificate of cause of death. (PDF 266 kb) [file 12874_2018_497_MOESM2_ESM.pdf]

INTERNATIONAL FORM OF MEDICAL CERTIFICATE OF CAUSE OF DEATH

**Cause of death**		**Approximate interval between onset and death**
**I**		
Disease or condition directly leading to death*	(a)	....
	due to (or as a consequence of)	
Antecedent causes Morbid conditions, if any, giving rise to the above cause, stating the underlying condition last	(b)	....
	due to (or as a consequence of)	
	(c)	....
	due to (or as a consequence of)	
	(d)	....
**II**		
Other significant conditions contributing to the death, but not related to the disease or condition causing it	....	....
	....	....
**This does not mean the mode of dying, e.g. heart failure, respiratory failure. It means the disease, injury, or complication that caused death.*		

(This form is extracted from *International statistical classification of diseases and related health problems. - 10th revision. Volume 2 - Instruction manual. Fifth edition*.

Further information on explanation of this form can be found at page 204-206 of this book *http://apps.who.int/classifications/icd10/browse/Content/statichtml/ICD10Volume2_en_2016.pdf.)*
