# Supplementary material for: Validation of verbal autopsy methods using hospital medical records: a case study in Vietnam
Source: BMC Med Res Methodol. 2018 May 18;18:43. doi: 10.1186/s12874-018-0497-7 (PMC5960129; doi:10.1186/s12874-018-0497-7)
Supplement: Supplementary file 3 — Validity by age groups. This supplementary document included two tables which describe the calculation of validity of the VA diagnosis on causes of death by age 25-69 years and 70+ years. (PDF 82 kb) [file 12874_2018_497_MOESM3_ESM.pdf]

Table 3.1. Validity of the VA diagnosis on causes of death by age 25-69 years

	ICD10 codes	True Positive	True Nagative	Medical records	Verbal Autopsy	Sensitivity 95% CI	Specificity 95% CI	PPV 95% CI
CANCER								
Lung cancer	C34	4	59	4	4	100	100	100
Liver cancer	C22	5	56	5	7	100	97 (92-100)	71 (38-100)
Colon and rectum cancers	C18-C21	2	61	2	2	100	100	100
Mouth and oropharynx cancers	C00-C14	3	59	4	3	75 (33-100)	100	100
Oesophagus cancer	C15	3	60	3	3	100	100	100
Stomach cancer	C16	1	62	1	1	100	100	100
Pancreas cancer	C25	2	61	2	2	100	100	100
CADIOVASCULAR DISEASES								
Stroke	I60-I69	4	53	6	8	67 (29-100)	93 (86-100)	50 (15-85)
Ischaemic heart disease	I20-I25	1	61	1	2	100	98 (95-100)	50 (0-100)
OTHER NON COMMUNICABLE DISEASES AND EXTERNAL CAUSES								
Diabetes mellitus	E10-E14	0	60	2	1	0	98 (95-100)	0
Cirrhosis of liver	K70, K74	1	58	4	2	25 (0-67)	98 (95-100)	50 (0-100)
Road traffic Injury	V01-V04, V06, V09-V80, V87, V89, V99	3	60	3	3	100	100	100
COMMUNICABLE DISEASES								
HIV/AIDS	B20-B24	2	60	3	2	67 (13-100)	100	100
Pneumonia J18	J12-J18	0	59	2	2	0	97 (92-100)	0
OTHER CAUSES		7		21	21			
**Total**		38		63	63			

Table 3.2. Validity of the VA diagnosis on causes of death by age 70+ years

	ICD10 codes	True Positive	True Nagative	Medical records	Verbal Autopsy	Sensitivity 95% CI	Specificity 95% CI	PPV 95% CI
CANCER								
Lung cancer	C34	4	48	4	4	100	100	100
Liver cancer	C22	1	51	1	1	100	100	100
Colon and rectum cancers	C18-C21	2	50	2	2	100	100	100
Stomach cancer	C16	1	51	1	1	100	100	100
CADIOVASCULAR DISEASES								
Stroke	I60-I69	10	35	12	15	83 (62-100)	88 (77-98)	67 (43-91)
Ischaemic heart disease	I20-I25	2	43	8	3	25 (0-55)	98 (93-100)	67 (13-100)
OTHER NON COMMUNICABLE DISEASES AND EXTERNAL CAUSES								
Diabetes mellitus	E10-E14	2	45	3	6	67 (13-100)	92 (84-100)	33 (0-71)
COPD	K70, K74	1	46	6	1	17 (0-46)	100	100
Cirrhosis of liver	J40-J44	1	51	1	1	100		100
Road traffic Injury	V01-V04, V06, V09-V80, V87, V89, V99	1	51	1	1	100	100	100
Communicable disease: Pneumonia	J12-J18	2	43	4	7	50 (1-99)	90 (81-98)	29 (0-62)
OTHER CAUSES		1	34	9	10			
**Total**		**28**		**52**	**52**			
